# Effectiveness of Equine-Assisted Activities and Therapies for Improving Adaptive Behavior and Motor Function in Autism Spectrum Disorder

**DOI:** 10.3390/jcm10081726

**Published:** 2021-04-16

**Authors:** Leonardo Zoccante, Michele Marconi, Marco Luigi Ciceri, Silvia Gagliardoni, Luigi Alberto Gozzi, Sara Sabaini, Gianfranco Di Gennaro, Marco Colizzi

**Affiliations:** 1 Child and Adolescent Neuropsychiatry Unit, Maternal-Child Integrated Care Department, Integrated University Hospital of Verona, 37126 Verona, Italy; leonardo.zoccante@aovr.veneto.it (L.Z.); marconimichele@icloud.com (M.M.); marco.ciceri@aulss9.veneto.it (M.L.C.); silvia.gagliardoni@gmail.com (S.G.); luigi.gozzi.1@gmail.com (L.A.G.); sara.sabaini@aovr.veneto.it (S.S.); 2 Corte Molon—ASD HorseValley, 37124 Verona, Italy; 3 Department of Pathology and Diagnostics, Integrated University Hospital of Verona, 37126 Verona, Italy; gianfradig@gmail.com; 4 Section of Psychiatry, Department of Neurosciences, Biomedicine and Movement Sciences, University of Verona, 37134 Verona, Italy; 5 Department of Psychosis Studies, Institute of Psychiatry, Psychology and Neuroscience, King’s College London, London SE5 8AF, UK

**Keywords:** hippotherapy, therapeutic horseback riding, atypical neurodevelopment, complementary and alternative methods, integrative interventions

## Abstract

Equine-assisted activities and therapies (EAAT) have been suggested to improve adaptive behavior, and possibly motor function, in autism spectrum disorder (ASD). This study investigated the effects of EAAT on adaptive behavior and motor function in 15 children with ASD (13 males) aged 7–15 years as well as the impact of EAAT on the magnitude of stress in the parent–child system and the evolution in the child interaction with both the trained therapist and the therapeutic animal through the 20 weekly sessions of EAAT. EAAT were associated with greater adaptive behavior and coordination (all *p* ≤ 0.01) as well as a progressive improvement in the child’s abilities to respond to the increasing complexity of such form of positive behavioral support (all *p* < 0.001). However, EAAT did not prove to be effective in reducing parental distress. Collectively, preliminary evidence presented here may have important public health implications and gives reason to hope that EAAT could possibly be an effective option in ASD, warranting further investigation of its potential benefits in clinical trials among larger samples.

## 1. Introduction

Autism spectrum disorder (ASD) includes a large and heterogeneous group of neurodevelopmental conditions diagnosed on the basis of a triad of behavioral impairments, including impaired social interaction, impaired communication and restricted and repetitive interests and activities [1], that can be observed prior to 3 years of age [2]. Research attempts to identify a single explanation for the three core aspects of ASD have found evidence of fractionation at the genetic, neural and cognitive levels [1]. Thus, ASD is by nature a multifactorial disease [3], whose different features are probably caused by different genes, associated with different brain regions and related to different core cognitive impairments, and are likely to respond to different types of treatment instead of a single “cure” or intervention [1]. Complementary and Alternative Methods (CAM) of treatment may support the classic medical approach [4]. In fact, it is not uncommon in the clinical practice that parents of children with ASD would ask for different treatments in addition to the pharmacological approach, including healthcare practices that traditionally have not been part of conventional medicine, especially when the child’s behavioral difficulties have not been adequately controlled. Complementary and integrative interventions have been suggested to promote interaction, communication, learning and motor skills [4]. Among the most implemented and effective types of CAM, we find swimming, art therapy, music therapy and equine-assisted activities and therapies (EAAT). EAAT incorporate hippotherapy, i.e., an integrated therapeutic program, and therapeutic riding, which originates from recreational activities. EAAT use a trained therapist and a therapeutic animal for Animal-Assisted Interventions (AAI) targeting specific childhood needs, especially in neuro-behavioral domains. EAAT take advantage of equine movement, which provides rhythmic movement to the person’s body [5]. Over the last decade, EAAT approaches have gained interest in ASD treatment in light of their potential improving effects on social functioning and postural control, which remain mostly refractory to medication [6].

A recent systematic review and meta-analysis of studies investigating the therapeutic effects of EAAT in children aged 3 to 16 years with ASD confirmed improvements in several domains, including socialization, engagement, maladaptive behaviors and problem-solving [6]. Of interest, EAAT were suggested to reduce stress [7], offering a possible biobehavioral explanation for the observed improvements in socio-cognitive functioning [8] and additional measures of well-being [9]. Further, the evidence of improvements also in motor coordination and posture [10] is of paramount relevance, in light of the high prevalence of poor motor proficiency in ASD [11], severe enough to constitute in some cases a motor coordination disorder [12] as well as the very limited availability of interventions targeting motor skills [13].

According to the current diagnostic criteria for ASD, the Diagnostic and Statistical Manual of Mental Disorders, 5th edition (DSM-5), conceptualizes three different severity levels, depending on the degree of social communication impairments and restricted repetitive patterns of behavior [2]. Importantly, independent evidence emphasizes that children with ASD have different symptomatologic trajectories over time as a function of their baseline impairment [14]. It is therefore plausible that ASD children with different levels of severity will present with different degrees of psychosocial, neurocognitive and neuromotor dysfunction, also responding differently to EAAT.

Using an exploratory approach, testing also for the effect of ASD symptom severity at baseline, the aim of this study was three-fold: (i) to conduct a comprehensive assessment of the effects of EAAT on psychosocial, neurocognitive and neuromotor abilities in children with ASD, (ii) to evaluate the impact of EAAT on the magnitude of stress in the parent–child system and (iii) to assess the evolution in the child interaction with both the trained therapist and the therapeutic animal through the animal-assisted activity sessions, by designing an ad hoc observation instrument.

## 2. Materials and Methods

### 2.1. Participants

Participants were recruited as part of a larger case-control study through convenience sampling, based on their willingness to participate, at the Veneto Autism Spectrum Disorder Regional Centre of the Integrated University Hospital of Verona, Italy. Children with suspected autism spectrum disorder (ASD) were screened with the Autism Diagnostic Observation Schedule, Second Edition (ADOS-2), the gold standard Level C measure of observational assessment for autism spectrum disorder [15]. ADOS-2 was administered and interpreted by appropriately credentialed professionals with completed clinical training and extensive prior experience with individuals with ASD. Diagnoses were made according to the Diagnostic and Statistical Manual of Mental Disorders, 5th edition (DSM-5), diagnostic criteria [2]. According to the DSM-5 severity level specifier, ASD children were categorized into 3 “severity levels” to help identify their support needs: level 3, requiring very substantial support; level 2, requiring substantial support; level 1, requiring support [2]. Exclusion criteria were (i) any significant medical illness, especially a neurological (e.g., cerebral palsy, epilepsy, or otherwise-classified motor handicap) or orthopedic (e.g., fracture, severe injury) condition, (ii) any previous relevant experience with horses and (iii) manifestation of distressed behavior such as to preclude the ability to complete the expected assessment (e.g., presenting significant risk to the child or those around them).

### 2.2. Ethics

Protocols and procedures were approved by the research ethics committee at the Integrated University Hospital of Verona (CESC 2242 and CESC 2243). Parents and guardians of all study participants were offered an extensive description of the study and then consented to their inclusion in the study as well as to the publication of data originating from it, by signing an informed written consent. The authors assert that all procedures contributing to this work comply with the ethical standards of the relevant national and institutional committees on human experimentation, with the Helsinki Declaration of 1975, as revised in 2013 [16]. 

### 2.3. Equine-Assisted Activities and Therapies

Equine-assisted activities and therapies (EAAT) were performed at the Italian Centre “HorseValley—Associazione Sportiva Dilettantistica” (Verona, Italy), between May and October 2020. Such amateur sports association was chosen as it had already been working with ASD children in the context of activities aimed at promoting social function in a playful context. Four professionals were directly involved in the EAAT: (i) a veterinarian expert in EAAT who tested the behavior and suitability of the animal for EAAT, (ii) a horse assistant who took charge of the horse during the sessions, monitoring the health and well-being of the horse, according to the instructions of the veterinarian to whom he reported any symptoms of horse distress, (iii) a healthcare professional (a Consultant Child and Adolescent Neuropsychiatrist) who acted as the EAAT project manager in coordinating the team in defining the objectives of the project, the related implementation procedures and the result evaluation, and (iv) a healthcare professional (a professional therapist), certified in Animal-Assisted Interventions (AAI) and with 11 years of experience with ASD individuals, who acted as the intervention representative in taking charge of the child during the session for the purposes of achievement of the project objectives as well as assuming responsibility for the correct horse–child relationship. With teamwork, three horses were deemed good candidates for EAAT in ASD individuals with different severity levels.

Each child underwent 20 individual weekly sessions of 45 min with the healthcare professional, in a space of 15 × 20 m. Also, 50% of sessions were individual sessions, and 50% were couple sessions. The number of individual therapeutic sessions and their duration was based on previous meta-analytic work (range: 30–180 min for 4–25 sessions) [6] as well as previous therapeutic experience of no clear advantages of longer and additional sessions in terms of mitigating the confounding effect of changes in the children’s attention span-persistence and environments (e.g., moving from school to vacation and vice versa). EAAT followed national guidelines for AAI (n. 60/CSR; 25 March 2015). Upon arrival, the child engaged in grooming, then activities on the ground were performed, followed by activities on the horse. In each session, different techniques and activities were proposed, with gradual difficulty and increasing complexity (from simpler to more complex tasks) and tailored to the child’s characteristics (respecting the child’s learning time), independently of their DSM-5 severity level specifier. Each activity was preceded by a verbal request and then followed by an example given by the intervention representative. The child tried to do the requested activity autonomously; if unable, the child was helped to perform the activity. A detailed description of tasks and exercises carried out is provided in the Supplementary Table S1.

### 2.4. Assessments

Both before (time 0, t0) and after the 20 individual sessions (time 1, t1), children’s psychosocial, neurocognitive and neuromotor abilities as well as stress in the parent–child system were assessed by a single rater, a trained clinical psychologist, using parent-report questionnaires. 

#### 2.4.1. Adaptive Behavior

Adaptive behavior was assessed using the Vineland Adaptive Behavior Scales—Second Edition—Survey Interview Form (Vineland-II), one of the most widely used standardized tests to measure an individual’s development of personal independence and social responsibility, by gathering information about day-to-day activities necessary to take care of oneself and to get along with others. The Vineland-II has established evidence of reliability and validity and has been used in many pediatric studies [17]. Items are rated on a five-point Likert scale across four domains: (i) communication, (ii) daily living skills, (iii) socialization and (iv) motor skills. The higher the score, the greater the child’s adaptive behavior [18]. 

#### 2.4.2. Neuromotor Function

Neuromotor function was assessed using the Developmental Coordination Disorder Questionnaire, as revised in 2007 (DCDQ’07), a widely used tool to measure motor skills in children. DCDQ’07 reliability and validity have been well-established [19], also in Italian populations of children [20]. Items are rated on a five-point Likert scale across three domains: (i) control during movement, (ii) fine motor and handwriting and (iii) general coordination. The higher the score, the greater the child’s coordination [21].

#### 2.4.3. Parent–Child Interaction

Parent–child interaction was assessed using the Parenting Stress Index–Short Form (PSI-SF), one the most widely used indices for parent–child system functioning. Reliability and validity of the test support its usefulness across diverse populations of different socioeconomic backgrounds [22]. Items are generally scored using a five-point scale across three domains: (i) parental distress, (ii) parent–child dysfunctional interaction and (iii) difficult child. The higher the score, the greater the parenting stress [23].

#### 2.4.4. Behavioral Progress Monitoring

In order to evaluate responsiveness to EAAT, the effect of such form of positive behavioral support and the attainment of individualized EAAT goals and objectives, the authors provided a conceptual overview for a criterion-referenced behavioral progress monitoring referred to as the Interaction Emotions Motor Skills (IEMS) observational scale, which was filled in at the end of every EAAT session, on the basis of behavioral progress observed during the entire session. Items are rated on a five-point scale across five specific areas of intervention: (i) social interaction, (ii) emotions-relation, (iii) behavior, (iv) gross motor skills and (v) fine motor skills. The higher the score, the greater the behavioral progress (Supplementary Table S2).

### 2.5. Data Analysis

Using an exploratory approach, a multilevel mixed-effects linear regression model was used to test for statistical significance in the association between the variables measured at the two time-points, by treating time as a binary variable (Vineland-II, DCDQ’07, PSI-SF; before the 20 individual sessions: time 0, t0; after the 20 individual sessions: time 1, t1). The same model was used to test for statistically significant changes across the 20 Interaction Emotions Motor Skills (IEMS) measurements, by treating time as a continuous variable. The level of significance was set at 0.05. Each model also tested for an effect of ASD severity on the outcome variables, by comparing Level 2 and Level 3 ASD children to Level 1 (least severe level) ASD children.

## 3. Results

Data were obtained on 15 children (13 males and 2 females) aged 7–15 years (mean (M) ± standard deviation (SD), 9.8 ± 2.2), 7 of whom with a Level 1 ASD, 6 with a Level 2 ASD and 2 with a Level 3 ASD. Table 1 reports descriptive statistics of all of the measurements performed both before (t0) and after the 20 individual sessions (t1). Table 2, Table 3 and Table 4 report the associations between the EAAT and the outcome variables (Vineland-II, DCDQ’07 and PSI-SF) as well as the association between the baseline severity and the outcome variables. There were significant differences due to time for all of the Vineland-II domains and the DCDQ’07 total score, but not for the PSI-SF total score. Finally, Table 5 reports the association between the EAAT and the IEMS sub-domains, indicating a progressive improvement as a function of time. A graphical representation of the association between the EAAT and the IEMS sub-domains is reported in the Supplementary Figures.

## 4. Discussion

This experimental study examined whether EAAT are effective in reducing difficulties associated with autism spectrum disorder (ASD). Results suggest that EAAT are associated with greater adaptive behavior and coordination as well as a progressive improvement in the child’s abilities to respond to the increasing complexity of such form of positive behavioral support. Interestingly, EAAT did not reduce parental distress, resulting paradoxically associated with a worsening in the parent-report of child’s temperament, defiance, non-compliance and demandingness. 

Most of the evidence regarding the effectiveness of EAAT in neuropsychiatric disorders with onset during neurodevelopment comes from studies conducted in children with cerebral palsy [6]. Instead, EAAT in ASD is relatively less investigated [6]. Also, while most studies investigating EAAT in ASD have tested for an effect of EAAT on behavior and environmental interaction, whose impairments represent the core symptoms to make a diagnosis of ASD, less evidence is available about the effect of EAAT for improving motor skills in ASD [24]. However, recent evidence highlights the need of recognizing motor impairments as one of the diagnostic criteria or specifiers for ASD as well as improving screening and intervention tools for motor function in ASD [25]. In particular, therapeutic programs involving activities with a horse have been successful in improving social functioning [8,9,10,26,27,28,29,30,31,32] and reducing aggressiveness [33]. 

More limited evidence indicates that ASD children receiving EAAT present with better stability and postural control [10], offering a promising therapeutic tool for what is a severe neurodevelopmental condition also in terms of postural instability [34]. However, studies specifically conducted among children and adolescents with a primary diagnosis of motor dysfunction or dyspraxia reported conflicting results. An earlier study found that an eight-week therapeutic riding intervention did not improve postural control and had only a small positive effect on gait performance [35]. Instead, a more recent study performed in a larger sample of similar age reported improvements across a number of gait parameters, possibly due to higher statistical power [36].

Our report adds to the previous literature converging on the effectiveness of EAAT in improving adaptive behavior and social functioning. Also, results support the clinical usefulness of EAAT in tackling coordination difficulties in ASD, encouraging the further investigation of such type of interventions in improving motor function in atypical neurodevelopment. As some outcome variables differed as a function of the ASD severity, further investigation is needed to fully address the effect of EAAT in larger samples of ASD individuals with different degrees of impairment.

The unexpected finding of a worsening in the sub-domain of the parent’s perception of the child’s behavior may have at least three explanations which are not mutually exclusive. First, aspects investigated by the PSI-SF “Difficult child” domain, that is the child’s behavioral characteristics and how they impact on the parents, may be stable phenotypic traits which show persistence even in the context of a clinically useful Complementary and Alternative Method (CAM) of treatment, in line with evidence for moderate phenotypic and etiological stability of autistic traits from childhood to early adulthood [37]. Second, EAAT, while being effective in improving adaptive behavior and motor function, do not represent an effective option to tackle parental distress. Third, as EAAT were expected to last for 20 sessions only, at the end of the therapeutic intervention, parents may have felt disappointed, especially if having high expectations towards the potential benefits of EAAT with reference to their concerns about the child’s growth and development, behavior and social issues, emotional wellbeing and independence in daily life. Future studies will have to clarify this issue, also in light of no evidence in the current study for a change in the total score of parenting distress.

The current study has to be seen in light of some strengths and limitations. The main strength of the study is that it included a broad range of outcome variables, including psychosocial, neurocognitive and neuromotor abilities, as well as a measure of stress in the parent–child dyad. The main limitations of the study are the absence of a control group and that its sample was too limited to fully investigate how the effectiveness of the EAAT would change as a function of the ASD severity. In particular, due to the one-group pretest–posttest study design, we cannot rule out that the observed changes in the outcome variables are not the result of the EAAT but due to alternative explanations such as changes unrelated to the intervention, including the maturation of the subjects as well as placebo and Hawthorne effects. Also, results may have suffered from employing an exploratory approach and ascribing linearity to the processes of sampling, data collection and data analysis. As we recruited children aged 7–15 years, it should be noted that the Vineland-II does not contemplate normative scores for the motor skills domain in subjects aged 7 and above. Moreover, the IEMS validity, reliability and variability remain to be tested. Finally, while extensively investigating the parent’s point of view, this study did not offer an evaluation of the effects of the EAAT in ASD from the clinician’s perspective. Preliminary results from this study require replication in larger populations from different socioeconomic backgrounds as well as before, during and after receiving other therapeutic interventions. Further, employing a longitudinal design, future studies may be able to investigate the potential presence of a differential response to EAAT in children with different degrees of ASD severity and whether the improvements observed after receiving EAAT do persist and for how long after discontinuation of the therapeutic intervention.

## 5. Conclusions

In summary, the study results suggest that equine-assisted activities and therapies (EAAT) may possibly be effective in improving adaptive behavior and motor function in autism spectrum disorder (ASD). Instead, evidence from this study does not support a role of EAAT in tackling parental distress. Collectively, this study may have important public health implications, as it gives reason to hope that Complementary and Alternative Methods (CAM) of treatment could possibly be effective in complex and multifactorial conditions such as ASD, in order to achieve the best possible lifetime outcome for individuals suffering from these conditions. However, while EAAT in this study was a one-time, time-limited, preliminary intervention that was possible thanks to the joint effort of a tertiary referral facility with extended experience in autism treatment and an amateur sports association with more than ten years of experience in EAAT, such intervention may be less feasible in the longer-term and in common clinical settings, due to infrastructure and resource costs.

## Figures and Tables

**Table 1:** Descriptive statistics of all of the measurements performed both before (t0) and after the 20 individual sessions (t1).

	Mean	SE	Min	Median	Max
Vineland-II Communication t0	48.1	6.5	20	49	105
Vineland-II Communication t1	57.5	6.4	20	59	96
Vineland-II Daily living skills t0	60.5	5.0	20	61	101
Vineland-II Daily living skills t1	72.5	5.2	28	73	109
Vineland-II Socialization t0	55.5	4.9	24	58	79
Vineland-II Socialization t1	63.0	5.4	21	69	91
Vineland-II Motor skills t0	66.9	8.3	20	90	103
Vineland-II Motor skills t1	83.6	6.9	20	94	112
DCDQ’07 total score t0	37.5	2.4	23	39	52
DCDQ’07 total score t1	40.2	2.1	25	41.5	52
PSI-SF Parental distress t0	30.5	2.4	16	32	44
PSI-SF Parental distress t1	30.1	2.6	13	31.5	41
PSI-SF Parent-child dysfunctional interaction t0	25.7	1.4	16	25.5	34
PSI-SF Parent-child dysfunctional interaction t1	26.2	1.9	16	27	40
PSI-SF Difficult child t0	29.6	1.7	20	30	39
PSI-SF Difficult child t1	32.4	1.9	20	33.5	42
PSI-SF total score t0	86.4	4.3	59	89	110
PSI-SF total score t1	87.7	6.0	52	92.5	112

SE, standard error; Min, minimum value; Max, maximum value; Vineland-II, Vineland Adaptive Behavior Scales—Second Edition—Survey Interview Form; DCDQ’07, Developmental Coordination Disorder Questionnaire, as revised in 2007; PSI-SF, Parenting Stress Index–Short Form; t0, time 0; t1, time 1.

**Table 2:** Association between the EAAT and the Vineland-II.

**Vineland-II Communication**	**Coefficient**	**95% CI**	***p*** **-Value**
Level 2 ^a^	−8.83	(−30.9; 13.2)	0.43
Level 3 ^a^	−39.00	(−70.7; −7.3)	0.02
EAAT	9.40	(4.8; 14)	<0.001
**Vineland-II** **Daily Living Skills**	**Coefficient**	**95% CI**	***p*** **-Value**
Level 2 ^a^	0.43	(−18.3; 19.1)	0.96
Level 3 ^a^	−20.82	(−47.8; 6.2)	0.13
EAAT	12.00	(8.3; 15.7)	<0.001
**Vineland-II** **Socialization**	**Coefficient**	**95% CI**	***p*** **-Value**
Level 2 ^a^	8.86	(−3.8; 21.5)	0.17
Level 3 ^a^	−36.89	(−55.1; −18.7)	<0.001
EAAT	7.47	(2.1; 12.8)	0.01
**Vineland-II** **Motor skills**	**Coefficient**	**95% CI**	***p*** **-Value**
Level 2 ^a^	26.29	(1.9; 50.6)	0.03
Level 3 ^a^	−11.21	(−46.3; 23.9)	0.53
EAAT	16.73	(6.1; 27.4)	<0.001

Vineland-II, Vineland Adaptive Behavior Scales—Second Edition—Survey Interview Form; CI, Confidence Interval; EAAT, Equine-Assisted Activities and Therapies; ^a^, as compared to level 1; the effect of the EAAT on the Vineland-II was tested by treating time as a binary variable, before the 20 individual sessions (time 0, t0) and after the 20 individual sessions (time 1, t1).

**Table 3:** Association between the EAAT and the DCDQ’07.

DCDQ’07	Coefficient	95% CI	*p*-Value
Level 2 ^a^	5.6	(−2.9; 14.1)	0.20
Level 3 ^a^	1.4	(−10.8; 13.6)	0.82
EAAT	3.6	(1; 6.3)	0.01

DCDQ’07, Developmental Coordination Disorder Questionnaire, as revised in 2007; CI, Confidence Interval; EAAT, Equine-Assisted Activities and Therapies; ^a^, as compared to level 1; the effect of the EAAT on the DCDQ’07 was tested by treating time as a binary variable, before the 20 individual sessions (time 0, t0) and after the 20 individual sessions (time 1, t1)

**Table 4:** Association between the EAAT and the PSI-SF.

**PSI-SF Parental Distress**	**Coefficient**	**95% CI**	***p*-Value**
Level 2 ^a^	−11.3	(−19.5; −3.1)	0.01
Level 3 ^a^	−1.3	(−12.4; 9.9)	0.83
EAAT	−0.4	(−2.5; 1.7)	0.69
**PSI-SF Parent–Child Dysfunctional Interaction**	**Coefficient**	**95% CI**	***p*-Value**
Level 2 ^a^	−4.7	(−10.8; 1.5)	0.14
Level 3 ^a^	−4.5	(−13; 3.9)	0.29
EAAT	0.5	(−1.4; 2.4)	0.62
**PSI-SF Difficult Child**	**Coefficient**	**95% CI**	***p*-Value**
Level 2 ^a^	−5.9	(−12.2; 0.3)	0.06
Level 3 ^a^	−2.2	(−10.7; 6.3)	0.62
EAAT	2.7	(0.2; 5.2)	0.03
**PSI-SF Total Score**	**Coefficient**	**(95% CI**	***p*-Value**
Level 2 ^a^	−21.6	(−38.7; −4.4)	0.01
Level 3 ^a^	−7.6	(−31.1; 15.9)	0.53
EAAT	1.3	(−4.5; 7.1)	0.67

PSI-SF, Parenting Stress Index–Short Form; CI, Confidence Interval; EAAT, Equine-Assisted Activities and Therapies; ^a^, as compared to level 1; the effect of the EAAT on the PSI-SF was tested by treating time as a binary variable, before the 20 individual sessions (time 0, t0) and after the 20 individual sessions (time 1, t1).

**Table 5:** Association between the EAAT and the IEMS sub-domains.

IEMS	Coefficient	95% CI	*p*-Value
Social interaction	0.32	(0.2; 0.4)	<0.001
Emotions-relation	0.31	(0.2; 0.4)	<0.001
Behavior	0.27	(0.2; 0.3)	<0.001
Gross motor skills	0.25	(0.2; 0.3)	<0.001
Fine motor skills	0.19	(0.1; 0.3)	<0.001

EAAT, Equine-Assisted Activities and Therapies; IEMS, Interaction Emotions Motor Skills; the effect of the EAAT on the IEMS sub-domains was tested by treating time as a continuous variable, across the 20 IEMS measurements.

## Data Availability

The data presented in this study are available on request from the corresponding author.

## Supplementary Materials

The following are available online at https://www.mdpi.com/article/10.3390/jcm10081726/s1, Table S1: Equine-assisted activities and therapies (EAAT); Table S2: The Interaction Emotions Motor Skills (IEMS); Figure S1: The figure shows the Interaction Emotions Motor Skills (IEMS) “Social interaction” sub-domain (*y*-axis) across the 20 individual sessions of equine-assisted activities and therapies (EAAT; *x*-axis); Figure S2: The figure shows the Interaction Emotions Motor Skills (IEMS) “Emotions-relation” sub-domain (*y*-axis) across the 20 individual sessions of equine-assisted activities and therapies (EAAT; *x*-axis); Figure S3: The figure shows the Interaction Emotions Motor Skills (IEMS) “Behavior” sub-domain (*y*-axis) across the 20 individual sessions of equine-assisted activities and therapies (EAAT; *x*-axis); Figure S4: The figure shows the Interaction Emotions Motor Skills (IEMS) “Gross motor skills” sub-domain (*y*-axis) across the 20 individual sessions of equine-assisted activities and therapies (EAAT; *x*-axis); Figure S5: The figure shows the Interaction Emotions Motor Skills (IEMS) “Fine motor skills” sub-domain (*y*-axis) across the 20 individual sessions of equine-assisted activities and therapies (EAAT; *x*-axis).
