# Supplementary material for: Effectiveness of Equine-Assisted Activities and Therapies for Improving Adaptive Behavior and Motor Function in Autism Spectrum Disorder
Source: J Clin Med. 2021 Apr 16;10(8):1726. doi: 10.3390/jcm10081726 (PMC8073280; doi:10.3390/jcm10081726)
Supplement: Supplementary file 1 [file jcm-10-01726-s001.zip › jcm-1131715-supplementary.pdf]

Journal of
Clinical Medicine

MDPI

Supplementary Table S1.. Equine-assisted activities and therapies (EAAT).

Interaction with the horse; spontaneous reciprocity; verbalization of parts of the horse; grooming activity with linear and circular movements, with one or two hands, then in opposite linear and circular directions	
Driving the horse on the ground with a lead shank along geometric paths, the same activity then proposed again riding the horse	
Get on the horse; field activities; riding figures	
Long reins with buttonhole in the halter - reins with carabiner hooked to the opening rein	
Lateral (raising right hand and right knee synchronously and vice versa) and cross-lateral exercises (raising right hand and left knee synchronously and vice versa)	
Lead the horse independently to the two operators placed on opposite sides of the field	
With four circles positioned at the corners of the field, the child directs the horse from one circle to another	
Synchronicity exercises (clapping following the rhythm of the horse's movement – with a bottom-up movement, based on the horse's gait – undulatory, jolting, rocking)	
Routes in autonomy (leading the horse independently, doing the slalom with the horse and interacting with it)	
Balance exercises	Open arms ("do the airplane")
	Standing (in the stirrups)
	Reverse saddling up
	Hold a stick (horizontal / vertical) and then change hands
	Riding the horse without stirrups
Spatial Games (going inside / outside of a drawn geometric figure)	
Riding bareback; hugged to the horse; lying on the horse	
Games with letters, images, and numbers; recognize / verbalize and connect	
Attribution of emotional states (looking at people and pictures of horses with similar emotional expressions)	
Grabbing or playing with objects while riding the horse	
Basketball - tennis on the horse: the child holds the horse with one hand and throws the ball with the other	
Throwing the ball with one hand and then switch hands	
Horses in pairs and two children hand in hand	
Individual reinforcement activities: balance-tonicity-synchrony-conduction	

Supplementary Table S2. The Interaction Emotions Motor Skills (IEMS).

		1	2	3	4	5
1	In the interaction with the adults, the boy / girl feels at ease					
2	In the interaction with the horse, the boy / girl shows signs of relax					
3	The boy's / girl interacts appropriately with respect to the situation					
4	The boy / girl experiences himself/ herself by taking initiative					
5	The boy / girl shows his/ her emotions through body language					
6	The boy / girl provides emotional feedback to the requests					
7	The boy / girl tolerates frustrations					
8	The boy / girl recognizes the playful dimension of the equestrian experience					
9	The boy / girl knows how to identify possible risks and dangers					
10	The boy / girl does not show consoling behaviors or stereotypies					
11	The boy / girl shows flexible and proportionate tone of voice					
12	The boy / girl has a space-time perception appropriate to the situation in terms of reckless behaviors, risk assessment, and experience of the flow of time					
13	The boy / girl has body movement patterns appropriate to the context					
14	The boy / girl controls naturally ipsilateral and contralateral skills					
15	The boy / girl can stay in dynamic balance on the horse in motion					
16	The boy / girl has a symmetrical and safe postural control					
17	The boy / girl uses correctly eye-manual coordination					
18	The boy / girl uses the reins according to the requests					
19	The boy / girl is able to grasp objects adequately according to the work environment					
20	The boy / girl is able to organize precision movements					

1 = never; 2 = rarely; 3 = occasionally; 4 = often; 5 = always; Social interaction: items 1-4; Emotions-relation: items 5-8; Behavior: items 9-12; Gross motor skills: items 13-16; Fine motor skills: items 17-20; sub-domain score range: 4-20; total score range: 20-100

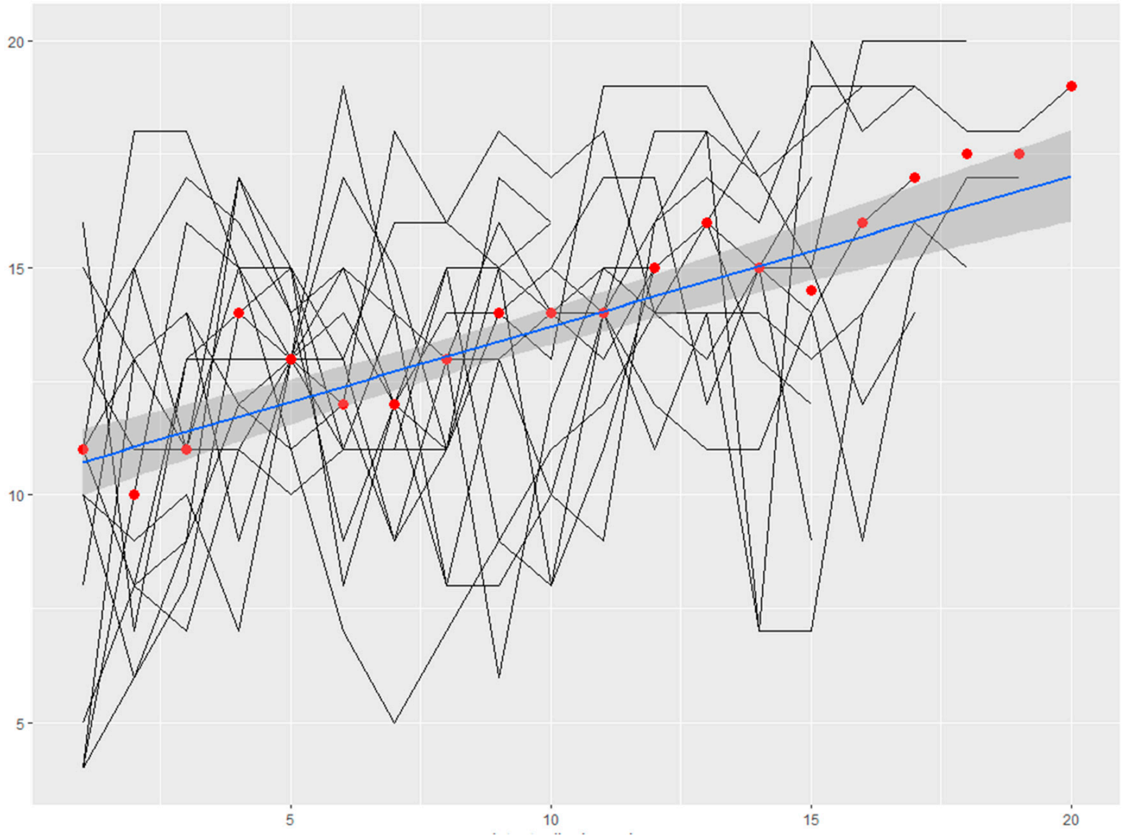

Supplementary Figure S1. The figure shows the Interaction Emotions Motor Skills (IEMS) "Social interaction" sub-domain (Y-axis) across the 20 individual sessions of equine-assisted activities and therapies (EAAT; X-axis); red circle markers show medians for every measurement; the blue line shows the regression.

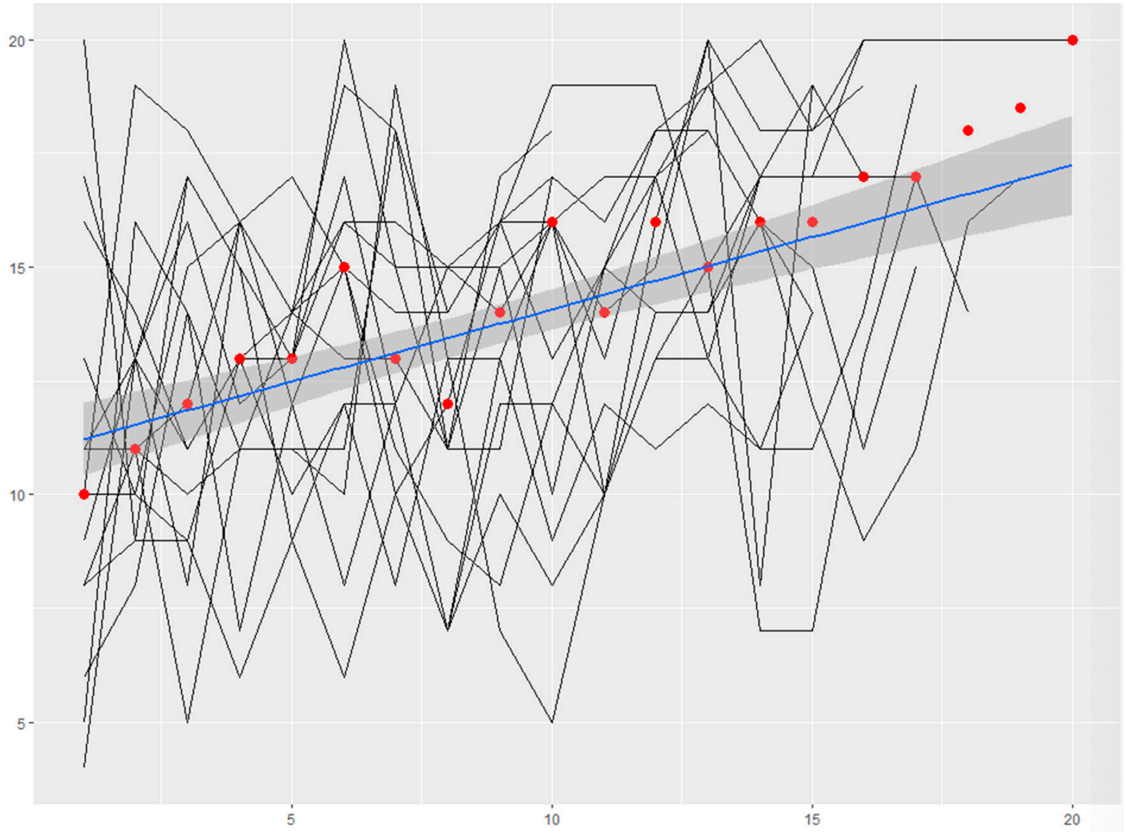

Supplementary Figure S2. The figure shows the Interaction Emotions Motor Skills (IEMS) "Emotions-relation" sub-domain (Y-axis) across the 20 individual sessions of equine-assisted activities and therapies (EAAT; X-axis); red circle markers show medians for every measurement; the blue line shows the regression.

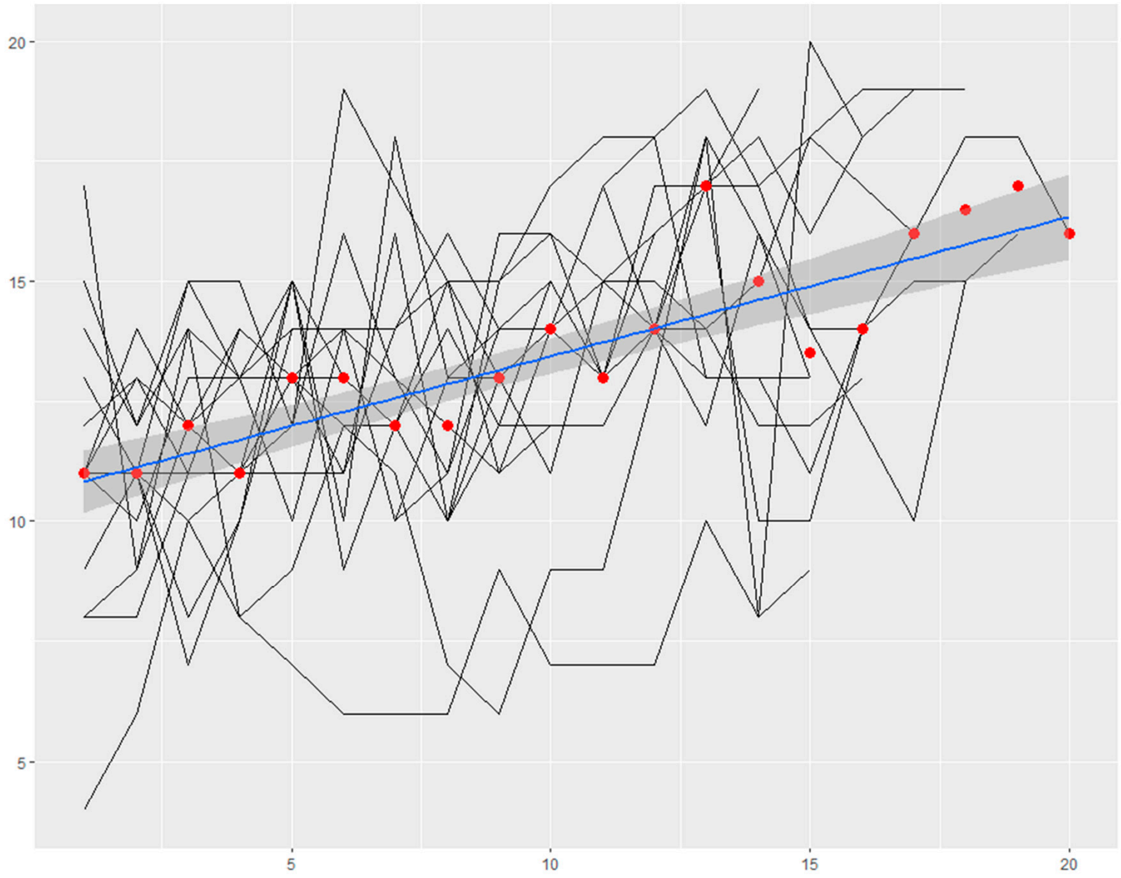

Supplementary Figure S3. The figure shows the Interaction Emotions Motor Skills (IEMS) "Behavior" sub-domain (Y-axis) across the 20 individual sessions of equine-assisted activities and therapies (EAAT; X-axis); red circle markers show medians for every measurement; the blue line shows the regression.

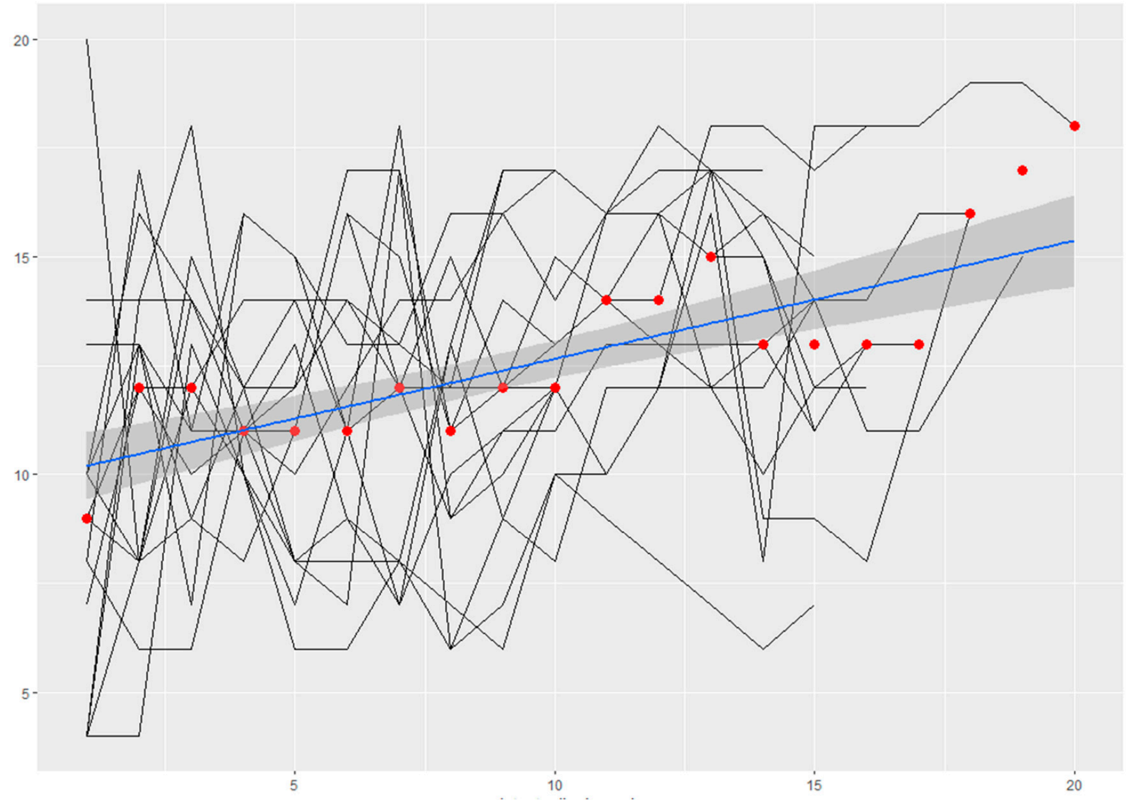

Supplementary Figure S4. The figure shows the Interaction Emotions Motor Skills (IEMS) "Gross motor skills" sub-domain (Y-axis) across the 20 individual sessions of equine-assisted activities and therapies (EAAT; X-axis); red circle markers show medians for every measurement; the blue line shows the regression.

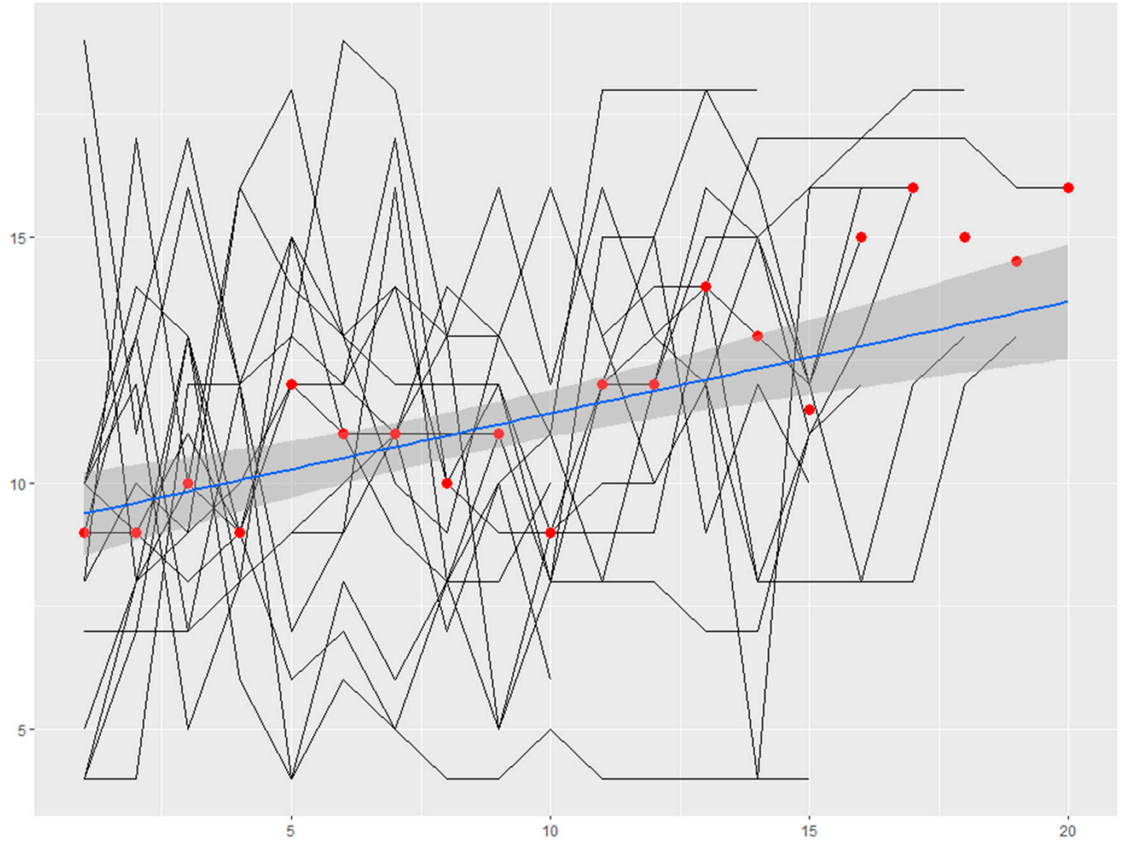

Supplementary Figure S5. The figure shows the Interaction Emotions Motor Skills (IEMS) “Fine motor skills” sub-domain (Y-axis) across the 20 individual sessions of equine-assisted activities and therapies (EAAT; X-axis); red circle markers show medians for every measurement; the blue line shows the regression.
